# Stabilization of after-traumatic flail chest by minimally invasive modified NUSS procedure in patient with starting respiratory insufficiency

**DOI:** 10.1186/s13019-023-02249-7

**Published:** 2023-04-11

**Authors:** Robert Chudzik, Krzysztof Buczyński, Paweł Rybojad, Marek Sawicki

**Affiliations:** 1 Department of Thoracic Surgery, Medical University of Lublin, Lublin, Poland; 2 Department of Thoracic Surgery, Holy Cross Cancer Centre in Kielce, Kielce, Poland

**Keywords:** Flail chest, Nuss procedure, nuss bar, Chest trauma, Case report

## Abstract

A flail chest is one of the possible medical conditions suffered by individuals who were injured in traffic accidents, caused by multiple fractures of the ribs and sternum. Which often results in paradoxical chest movements. The consequence may be respiratory failure and need for long-term mechanical ventilation. Such treatment require Intensive Care Unit and may be associated with the possibility of numerous complications.

Modified Nuss procedure was performed in 79-year-old man, a victim of a car crash to obtain stabilization of the flail chest. After compensation of paradoxical movements on the third day it was possible to end mechanical ventilation. A quick procedure dedicated to the congenital deformation of the chest made it possible to avoid long, expensive intensive therapy with possible respiratory complications.

The NUSS procedure enables the effective and safe treatment of a flail chest in a selected group of patients.

In our department treatment of pectus excavatum is performed with the use of both the NUSS and Ravitch procedures. Broken ribs and flail chest are treated both conservatively and surgically by performing rib anastomoses with the “Matrix rib” set. The presented case of modified Nuss procedure to heal the flail chest has been performed for the fist time in our department. Until now, the standard treatment of such patients has been internal stabilization with positive end expiratory pressure (PEEP) ventilation therapy sometimes assisted by surgical procedure with the use of “Matrix rib” set. Long-term, expensive treatment with the possibility of numerous complications. Our study aims to show both the possibilities of effective treatment of this type of injuries and to draw attention to the significant saving of both financial and human resources by shortening hospitalization in the ICU [1–4].

Case:

A 79-year-old man was transported by helicopter to our hospital after a car accident. Trauma CT revealed: head without traumatic lesions, chest: anterior mediastinal hematoma, no pneumothorax or hemothorax. Fracture of the manubrium and body of the sternum, fracture of the right ribs II-VII (V and VI with displacement, probably also VIII and IX, left II-VI (II with displacement). Small foci of pulmonary parenchyma contusion. Abdominal cavity and pelvis - no traumatic lesions. Fractures of the right wrist and left ankle. Glasgow Coma Scale: 15, circulatory and respiratory efficiency (oxygen therapy, saturation 98%), visible paradoxical respiratory track, features of the anterior chest window fractures, palpation pain in the area of ​​the sternum and on both sides of the costal arches.

On admission to ER Arterial Blood Gas test was performed: pO2: 26.6 mmHg, pCO2 45.1 mmHg.

Initially, patient was treated with passive oxygen therapy (95% saturation).

The next day, arterial blood gas test showed: pO2 69mmHg, pCO2 39mmHg. Due to huge paradoxical movements there was a high risk of increasing parameters of respiratory failure. The patient was qualified for the surgical stabilization of the anterior chest window for the next day (Fig. 1).

**Fig. 1:**
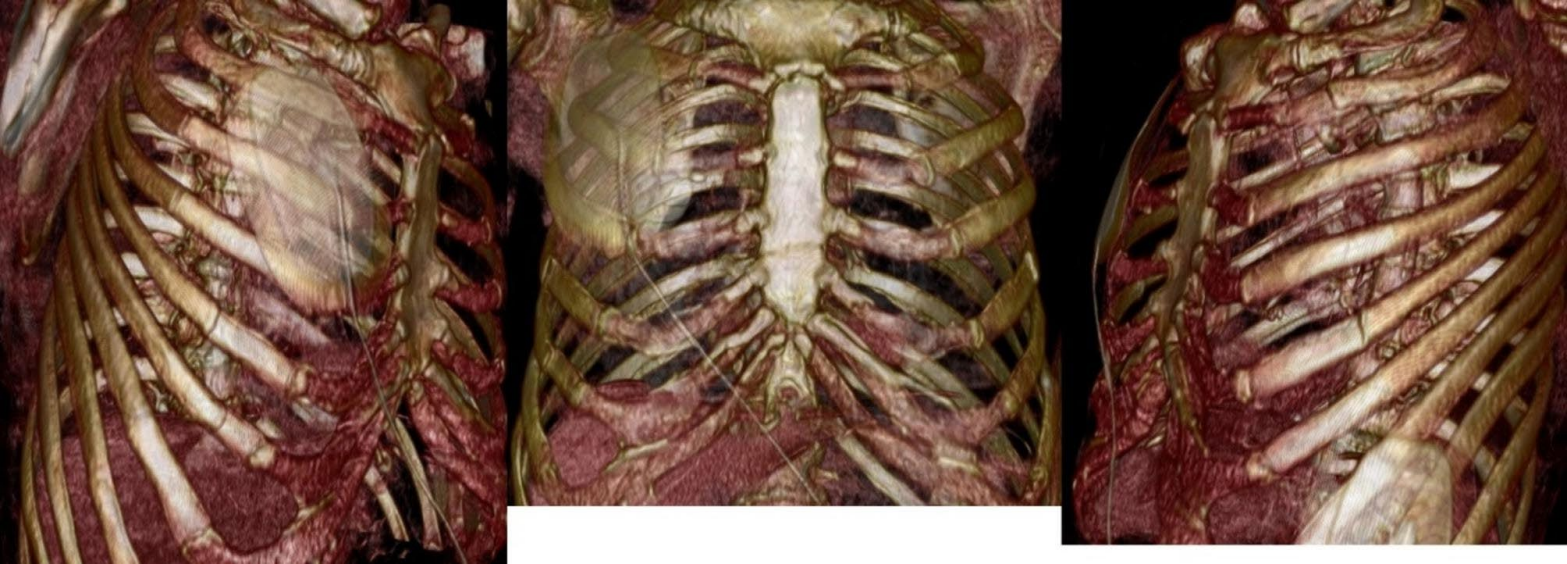
CT scans before surgery

Because of many bone fractures in the anterior part of the chest, stabilization with matrix system seemed to be insufficient. Therefore we decided to use modified Nuss procedure. We cut about 2.0-cm incision on both sides of the chest, corresponding to the fifth intercostal spaces on both sides. Additional port for camera was introduced on right side – where after surgery drain was placed (thorax fi 28). Submuscular tunnels were made from each incision to a position lateral to the flail segment. A introducer bar was passed from a pre-selected uninjured intercostal incision, through the submuscular tunnel, and into the pleural cavity under thoracoscopic monitoring to other side of sternum. The Nuss bar was passed by the runner tape across the mediastinum to the other side of the flail chest. The bar was rotated 180 degrees so that the convexity of the bar faced anterior to support the flail sternum. The Nuss bar was fastened to a rib on the right side – in order to maintain its stabilization.

After the procedure, the patient was transferred to the Intensive Care Unit for further treatment. He was ventilated in VC mode with FiO2 = 0.4. Chest ultrasound showed expansion of both air lungs with merely no fluid collection within both pleural cavities.

On the next day parameters of respiratory ventilation could be corrected to FiO2 = 0,3 due to improvement in respiratory efficiency. No signs of chest instability were observed, even in shallow sedation. On 3rd day after the surgery, the patient was extubated. Chest showed correct breathing mechanics with no signs of unstable chest.

Next day, the chest tube was removed. Control x-ray of the chest showed reexpantion of lung parenchyma, no signs of fluid collection, correct position of the NUSS bar, stabilized fractures of chest bones. Patient did not need additional oxygen therapy.

Due to the persistent haematuria a diagnosis was performed to reveal the presence of a bladder tumor. The patient did not agreed on surgical treatment. Because of the performed diagnostics procedures, hospitalization times was significantly extended.

Due to the advanced neoplastic process of the urinary bladder, the removal of the NUSS plate was not planned. The patient was discharged home with the recommendations of periodic thoracic control. However, within one month of the end of hospitalization, the patient was admitted to hospital due to a heart attack, which was finally the cause of his death. Pathologist did not find any connection between Nuss bar stabilization and heart disorders (Fig. 2).

**Fig. 2:**
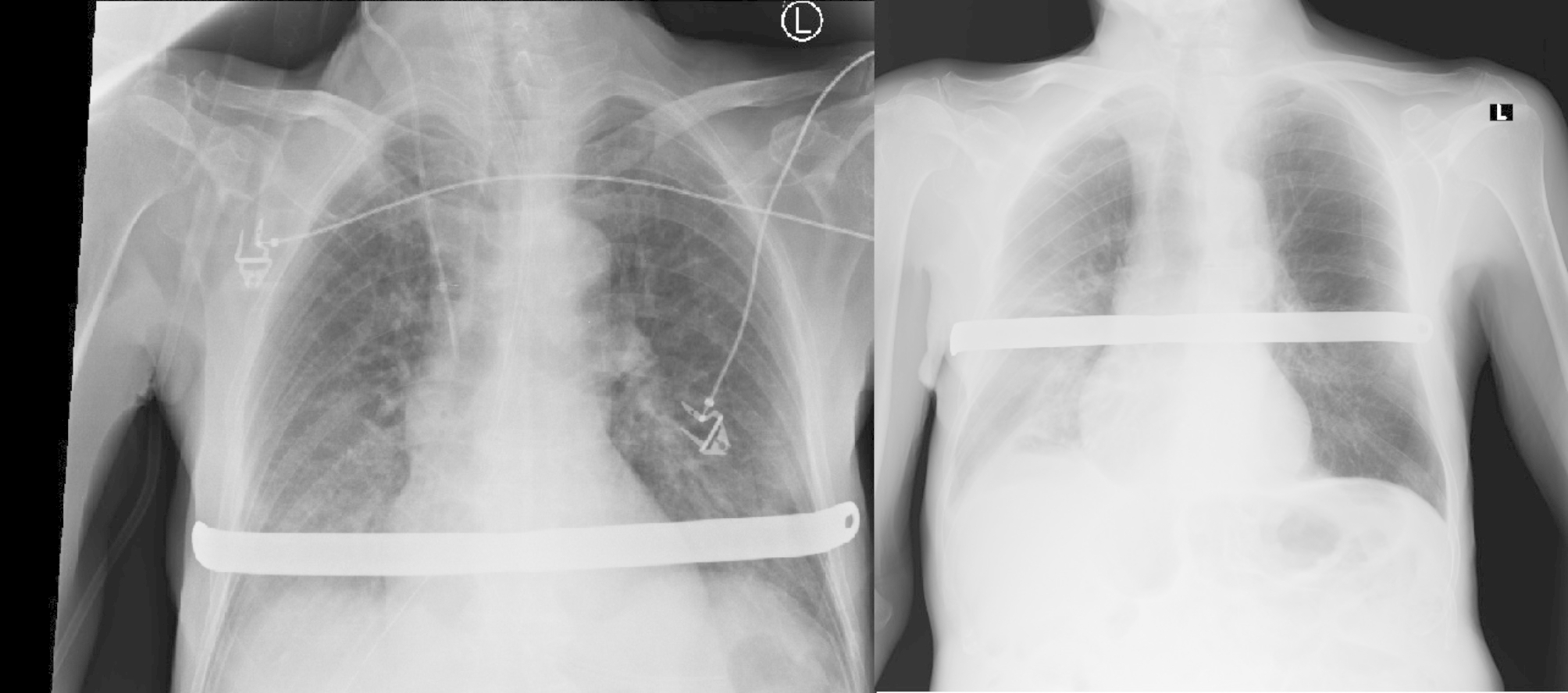
X-ray day after surgery and day of discharge

## Conclusion

The best rating of effectiveness of described procedure was the possibility of completion mechanical ventilation on the 4th day after surgery. This reduces the need for prolonged positive end expiratory pressure ventilation as well as long hospitalization at the ICU.

Because of the long stay of the patient in the hospital (30 days) due to problems related to hematuria, we had the opportunity to closely follow the after operation period. The patient did not report any problems related to the trauma of the chest.

We believe that modified NUSS procedure can be considered as a way of flail chest treatment in selected group of patients. The simplicity and minimally invasiveness of the procedure emphasize this statement. Removing the NUSS bar in the future should also be considered as minor problem.

It should be noted that our patient was an ideal candidate for this procedure: no additional diseases in medical history, isolated chest trauma with paradoxical movements and minor fractures of long bones. Nevertheless, we believe that described procedure may be indicated even in more complicated cases of flail anterior chest wall. Whether flail chest occured in the effect of trauma or other type of injury (for example post-CPR status), stabilization should be considered, especially when paradoxical movements can be observed. In the medical database we found some information about similar surgeries performed. Due to the simplicity and possible positive effects for a selected group of patients, a prospective multicenter study should be planned in order to draw wider conclusions and possible standardization.
